# Following the Flood

**DOI:** 10.3201/eid3010.AC3010

**Published:** 2024-10

**Authors:** Byron Breedlove

**Affiliations:** Centers for Disease Control and Prevention, Atlanta, Georgia, USA

**Keywords:** vector-borne infections, viruses, mosquitoes, Alfred Sisley, Flood at Port-Marly, art–science connection

**Figure:**
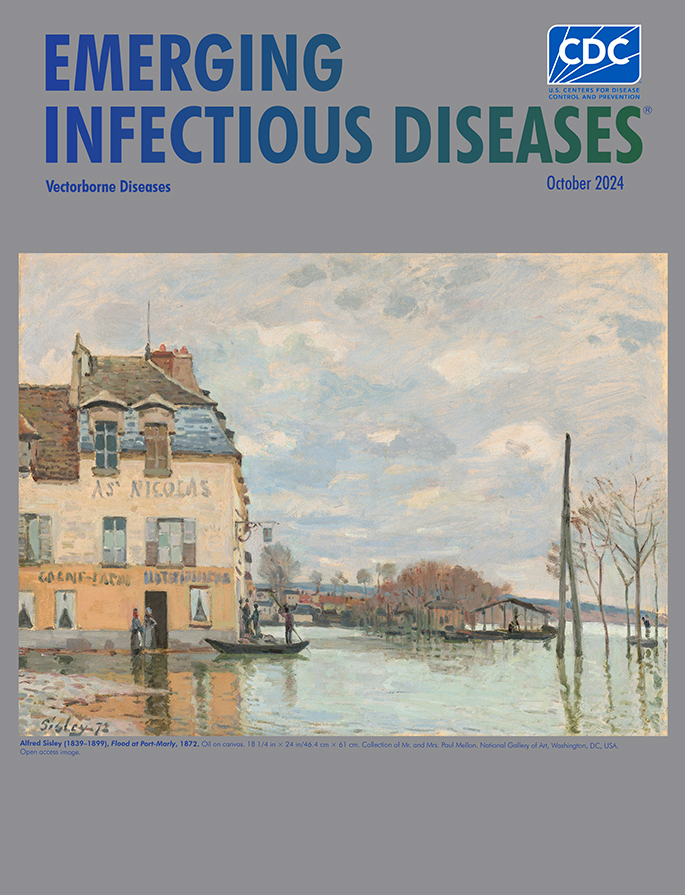
**Alfred Sisley (1839–1899). *Flood at Port-Marly*, 1872.** Oil on canvas, 18¼ in x 24 in/46.4 cm x 61 cm. Collection of Mr. and Mrs. Paul Mellon. National Gallery of Art, Washington, DC, USA. Open access image.

“Great floods have flown from simple sources.”—William Shakespeare, *All’s Well that Ends Well*, Act 2, Scene 1, line 142

Flooding is the most frequently occurring natural disaster worldwide. According to the data portal Statista, 164 floods were reported in 2023, and no doubt others occurred. Hydrologists categorize the most common types of flooding as coastal floods, river floods, storm surges, and flash floods. Regardless of whether it’s a roiling torrent or a seemingly languid flow, floods can damage and destroy cities, towns, building, bridges, and roads; decimate natural landscapes, farms, and crops; displace human and animal populations; disrupt commerce, travel, and healthcare; and cause loss of life during the flooding and afterwards.

This month’s cover features *Flood at Port-Marly*, an event French impressionistic artist Alfred Sisley witnesses and recorded. Biographies of Sisley note that he was born in Paris, France, and his parents were British expatriates. He lived in France and occasionally visited England. In 1862, Sisley received training at the Paris studio of Swiss artist Marc Gabriel Charles Gleyre, where he met Claude Monet, Pierre Renoir, and Frederic Bazille. The four became friends, sharing studio space and exchanging ideas, and are credited as being among the originators of Impressionism. Despite his associations with other Impressionistic artists and a portfolio of around 900 oil paintings, 100 pastels, and numerous drawings, Sisley received little recognition and lived much of his life in poverty.

The Getty Museum says of Sisley: “A pure landscape painter unconcerned with the challenge of history painting, he celebrated the intimate qualities of the places he lived in, exploring the effects of changing light and weather and mapping scenes from a variety of viewpoints in different seasons.” The U.S. National Gallery of Art, home to this work, notes that “Flooding early in the spring of 1872 drew Sisley to Port-Marly, a village on the Seine near Louveciennes, the artist’s home. The water here is calm and human activity is minimal. Rather than dramatic or picturesque incident, the artist’s attention was engaged by purely visual effects of rain-laden clouds and water-covered streets.”

In this traditionally composed painting, eddying, pink-tinted clouds float over gently rippling water. The river Seine has spilled over its banks, covered the street, and stranded people. There is no sense of eminent danger from surging floodwaters nor havoc and horror following a ruinous deluge. The water has either just stopped short from or receded from the entrances of the two-toned building. The National Gallery of Art offers relates, “Two women in long skirts stand at a darkened, open doorway near the front corner of the structure. A sign on the side of the building hangs from a horizontal arm over three men in and near a shallow boat, which is being propelled by a man who stands in the stern with a long stick. The street is so wet that it first appears to be a canal or river. It is only when we notice dashes of mauve, pale pink, and gray to our left that we realize the cobblestone road is flooded.”

This village has apparently escaped direct devastation from flooding, but indirect effects might still occur. Accumulations of standing water that linger could increase the risk for transmission of various vectorborne diseases, such as malaria, dengue fever, West Nile virus disease, and yellow fever. However, although floodwaters could create ideal breeding grounds for mosquitoes and other arthropod vectors in some circumstances, heavy flooding might also wash away larval breeding habitats, decreasing the population of adult mosquitos.

The relationship between vectorborne infections and flooding is not cut and dried. The dynamics of transmission for such diseases are complex and depend on interactions of multiple biologic and environmental factors, adding to the challenges hampering public health efforts to predict, prevent, and respond to outbreaks of vectorborne diseases that may follow flooding.
